# A dataset describing brooding in three species of South African brittle stars, comprising seven high-resolution, micro X-ray computed tomography scans

**DOI:** 10.1186/s13742-015-0093-2

**Published:** 2015-11-17

**Authors:** Jannes Landschoff, Anton Du Plessis, Charles L. Griffiths

**Affiliations:** 1 Department of Biological Sciences and Marine Research Institute, University of Cape Town, Rondebosch, South Africa; 2 CT Scanner, Central Analytical Facility, Stellenbosch University, Stellenbosch, South Africa

**Keywords:** Micro CT, Ophiuroids, Scanning, Imaging, Anatomy, Morphology, Brood care, μCT, Brittle stars

## Abstract

**Background:**

Brooding brittle stars have a special mode of reproduction whereby they retain their eggs and juveniles inside respiratory body sacs called bursae. In the past, studying this phenomenon required disturbance of the sample by dissecting the adult. This caused irreversible damage and made the sample unsuitable for future studies. Micro X-ray computed tomography (μCT) is a promising technique, not only to visualise juveniles inside the bursae, but also to keep the sample intact and make the dataset of the scan available for future reference.

**Findings:**

Seven μCT scans of five freshly fixed (70 % ethanol) individuals, representing three differently sized brittle star species, provided adequate image quality to determine the numbers, sizes and postures of internally brooded young, as well as anatomy and morphology of adults. No staining agents were necessary to achieve high-resolution, high-contrast images, which permitted visualisations of both calcified and soft tissue. The raw data (projection and reconstruction images) are publicly available for download from GigaDB.

**Conclusions:**

Brittle stars of all sizes are suitable candidates for μCT imaging. This explicitly adds a new technique to the suite of tools available for studying the development of internally brooded young. The purpose of applying the technique was to visualise juveniles inside the adult, but because of the universally good quality of the dataset, the images can also be used for anatomical or comparative morphology-related studies of adult structures.

## Data description

### Background and purpose of data collection

Brooding and live-bearing of well-developed offspring is a rather uncommon phenomenon in ophiuroids (brittle stars). However, some species brood young in special brood chambers termed bursae [1]. For a long time, dissection has been the only way to count and measure internally brooded young and determine their position inside the adult, but micro X-ray computed tomography (μCT) has several advantages over the historical approach [2]. For brooding brittle stars the non-destructive nature of the technique allows an in situ view of juveniles in an undisturbed sample. After the scan, not only is the sample still intact, but also the available dataset can be disseminated for further analyses and repetition of the study [3]. This dataset was created with the purpose of comparing brooding adult and brooded young brittle stars among different species and to visualise the juveniles in reconstructed three-dimensional (3D) images. An accompanying publication presents the results of this analysis [4].

### Specimens scanned

The seven brittle star scans presented in this dataset comprise five individuals of three species. Members of two families were scanned: one specimen of *Amphipholis squamata* (Delle Chiaje, 1828), and one specimen (in two scans) of *Amphiura capensis* Ljungman, 1867 (Family Amphiuridae, collected at Mouille Point, Cape Town, South Africa, GPS position S33°53.932’ E18°24.573’), as well as four specimens of *Ophioderma wahlbergii* Müller and Troschel, 1842 (Family Ophiodermatidae, collected at Windmill Beach, False Bay, South Africa, GPS position S34°12.046’ E018°27.397’). While the amphiurids *A. squamata* and *A. capensis* are small species of disc diameter 5–10 mm, *O. wahlbergii* is an unusually large brooding species with >30 mm disc diameter [5]. Pregnant individuals were selected for during sampling. All samples were freshly fixed in 70 % ethanol and were scanned in air and without any further treatment. Nevertheless, we included a scan trial of *O. wahlbergii*, which did not contain any juveniles and was probably a male. All other scanned specimens contained brooded young. With the exception of *A. capensis*, and in order to increase the isotropic voxel resolution, only the disc of the animals, which holds all the body organs and the brooded young, was scanned. The arms of *O. wahlbergii* and *A. squamata* were clipped to avoid the sample wobbling during scanning. However, we later found that our concerns about sample movement due to long, unstable arms were baseless-therefore the data for *A. capensis* contain two scans: one full scan including the arms (14 μm isotropic voxel size) and one scan of only the disc, but where the arms were not cut off the body (5.4 μm isotropic voxel size). These two scans of the same individual can be overlaid to gain both high resolutions of the body and the entire field of view in the whole sample. The *A. capensis* specimen, museum ID MB-A066817, was deposited in the Iziko Museums of South Africa, Cape Town.

### Scanning, data processing and quality control

Micro-CT scans were performed using two systems at the Stellenbosch University CT Scanner Facility; the General Electric Phoenix V|Tome|X L240 with NF180 option, and the General Electric Phoenix Nanotom S. Samples had to be rigid to ensure no movement during scanning and were placed, one at a time, on top of a plastic rod with dense polystyrene foam as a platform. Depending on sample geometry, the sample was attached to the top of the platform by thin, double-sided tape, or within a hollow cut in the polystyrene foam. Settings were chosen so that X-ray spot size did not exceed the selected scan resolution, providing good X-ray penetration as indicated by high transmitted brightness values in the live digital X-ray images while setting up X-ray scan parameters (X-ray voltage from 60 to 100 kV and current from 100 to 200 μA in the various scans). Furthermore, background detector calibrations before each scan, as well as visual inspections of the reconstruction images, ensured high data quality and good image contrast. To reduce potential beam hardening artefacts a 0.1 mm copper beam filter was used in the 100 kV scans. The 60 kV scan used no filter. Lower voltage allows better material discrimination, while potentially increasing unwanted image artefacts [6]. In this case no such artefacts were present and quality of the scans was adequate. In each 360° rotation 1600–3100 images were taken; image acquisition was 500–1000 ms with no averaging of images. Acquired projection images were reconstructed using system-supplied General Electric Datos reconstruction software. Additional functions were also used, such as correcting for rotation axis offset and making use of a region of interest in each image to correct for X-ray intensity fluctuations. The reconstructed dataset was subsequently analysed by Volume Graphics VGStudioMax 2.2, though image stacks were generated to be compatible with other software packages.

### Potential uses

**Fig. 1:**
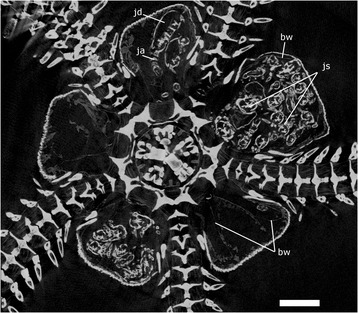
Micro-CT scanning image of disc of *Amphiura capensis*. The 5.4 μm isotropic voxel size scan shows calcified skeleton components (intensely bright areas), juveniles (js; arm = ja, disc = jd) and soft tissues such as the bursal wall (bw). Scale bar: 1 mm

The presented dataset can be used for in situ examination of brooded juveniles inside the bursae of adult brittle stars, but at the same time the data contain a complete suite of anatomic and volumetric information on both adults and juveniles, which can be used for morphological and/or systematic comparisons [7]. Not only does the quality of the data allow identification of calcified structures, but examinations of the soft tissues and inner organs are also possible (Fig. 1).

### Recommended software for visualising data

Individual X-ray projection images and CT slice images can be opened with image viewing software, e.g., Irfanview or ImageJ. The full 3D dataset can be opened by any 3D data software by importing the image stack. Some examples are ImageJ, Avizo, Simpleware, Octopus or VGStudioMax. Additionally, a free 3D viewer for Windows, called myVGL, can be requested from Volume Graphics [8]. The PCA file can be opened in a text reader e.g., notepad. When opening a full 3D dataset, the entire volume is loaded to memory and, depending of the volume size, enough memory is required. We recommend an operating system with at least 16 GB RAM.

## Availability of supporting data and materials

### Data availability

The presented dataset is deposited in the *GigaScience Database* repository [9]. Each scan contains two folders: one for two-dimensional X-ray projection images and one for the stack of reconstructed slice images. The X-ray projection images are those directly from the scanner and can be used for reconstruction of the volume data. The stack of reconstructed slice images comprise the volume data of the region of interest and can be read by any software package; slice images can also be simply viewed in any image viewer program. In addition, each scan folder contains a PCA file with the respective scan settings.

## Abbreviations

3D: Three-dimensional
CT: Computed tomography
DOI: Citable digital object identifier
GB: Gigabyte
μCT: Micro computed tomography
PCA: Phoenix CT acquisition
RAM: Random access memory.
